# Exploring Diagnostic Complexities: A Case Report of Osteitis Condensans Ilii and Osteitis Pubis

**DOI:** 10.7759/cureus.67543

**Published:** 2024-08-22

**Authors:** Devyansh Nimodia, Ravishankar Patil, Pratapsingh Parihar, Sakshi S Dudhe, Paritosh N Bhangale, Rishitha Kotla

**Affiliations:** 1 Radiodiagnosis, Jawaharlal Nehru Medical College, Datta Meghe Institute of Higher Education and Research, Wardha, IND; 2 Psychiatry, Jawaharlal Nehru Medical College, Datta Meghe Institute of Higher Education and Research, Wardha, IND

**Keywords:** osteitis condensans ilii, postpartum, osteitis pubis, noninflammatory, osteopathia condensans ilii, sacroiliac joint

## Abstract

Osteitis condensans ilii (OCI) is a benign etiology of lumbago, characterized by its self-limiting nature. Referred to as hyperostosis triangularis ilii, this condition is a rare occurrence, primarily manifesting in the female demographic. The etiology of this ailment remains undisclosed. Predominantly observed in the vicinity of the ileum, it may be erroneously interpreted as the involvement of the sacroiliac joint. Not characterized by inflammation, this disorder commonly emerges as a postpartum sequela in females. Osteitis pubis is a constrained inflammatory disorder characterized by pain affecting the pubic bones, joints, and associated tendons. This condition has been documented following instances of trauma, pelvic surgical procedures, childbirth, excessive athletic activity, and certain rheumatic ailments. We present a case of bilateral OCI with osteitis pubis in a 21-year-old female who presented with persistent lumbago as evidenced by radiological findings. Magnetic resonance imaging (MRI) corroborated the diagnostic procedures, indicating the presence of OCI with osteitis pubis. Sclerotic fibrosis exhibited a distinctly delineated and compact appearance on MRI, facilitating its differentiation from other pathologies linked to back pain. Regrettably, there exists no definitive remedy for these conditions, with lifestyle adjustments representing the sole measure that may aid in preventing disease recurrence.

## Introduction

Osteitis condensans ilii (OCI) is a benign etiology frequently implicated in the pathogenesis of persistent axial lumbosacral pain. Also referred to as osteopathia condensans ilii or hyperostosis triangularis ilii, OCI is typified by benign, triangular sclerosis affecting the ilium adjacent to the sacroiliac joint [1]. This case presents bilateral iliac sclerosis alongside OCI, accompanied by a brief literature review. Sicard, Gally, and Haguenau first described OCI in 1926 [1]. OCI is typically detected as an incidental radiographic finding demonstrating ileal sclerosis. Predominantly occurring in women of childbearing age during the antepartum or postpartum periods, OCI may also manifest in nulliparous females and males [2]. Often misidentified as iliac osteoarthritis, OCI generally lacks symptoms, although sporadic instances may present as early-onset lumbosacral pain resembling axial spondyloarthropathy [3]. Diverging from osteoarthritis, OCI typically conserves joint space, exhibits non-progressive behavior, and frequently lacks abnormal laboratory findings despite occasional overlapping clinical features [4]. Differential diagnosis of conditions involving the sacroiliac joints necessitates consideration [5]. The occurrence of OCI with osteitis pubis represents a seldom-reported uncommon complication. Symptoms of osteitis pubis encompass pelvic discomfort, which may extend to the inner thigh or lower abdomen and is exacerbated by abdominal straining or movement (particularly standing up or ascending stairs) [6]. Physical examination reveals tenderness around the pubis, potential joint instability, and sporadic fever [6]. Blood examinations exhibit features of an inflammatory process, which can be corroborated through bone scanning, as demonstrated in our patient [6].

## Case presentation

**Figure 1:**
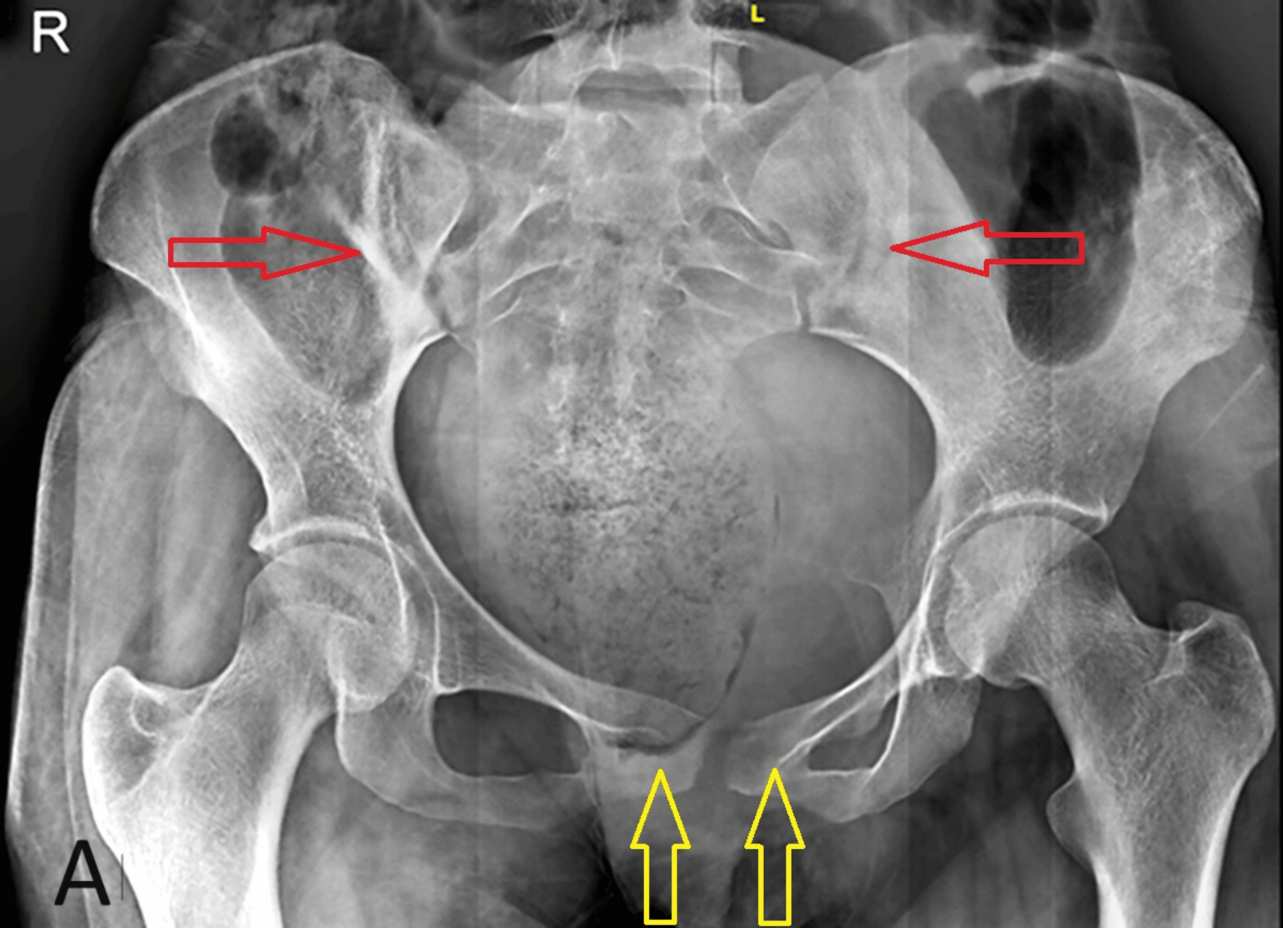
X-ray revealing subchondral sclerosis and bony margin irregularities in the bilateral pubic symphysis (yellow arrow) and the bilateral sacroiliac joint (red arrow), more on the right side.

A 30-year-old married woman presented at our clinic with a prolonged history of persistent lumbar pain over two years. She reported experiencing back discomfort during her initial peripartum phase, which persisted thereafter. The pain was localized primarily at the L4-L5 vertebra in the lower back midline and was characterized by gradual and progressive onset, evolving from mild to severe intensity. The patient described the pain as a continuous, dull ache lasting for two to three days with a one-week gap, exacerbating during menstruation and radiating to various areas such as the gluteal region, lateral thigh, and back. Aggravating factors included bending and lifting heavy objects, while pain relief was achieved through medication. Physical examination revealed no abnormalities, and diagnostic imaging indicated significant sclerosis at the iliac border of sacroiliac joints and pubis. Subsequent tests ruled out inflammatory conditions, and an X-ray and magnetic resonance imaging (MRI) investigation was advised. An X-ray revealed subchondral sclerosis and bony margin irregularities in pubic symphysis and the bilateral sacroiliac joint, more on the right side (Figure 1).

**Figure 2:**
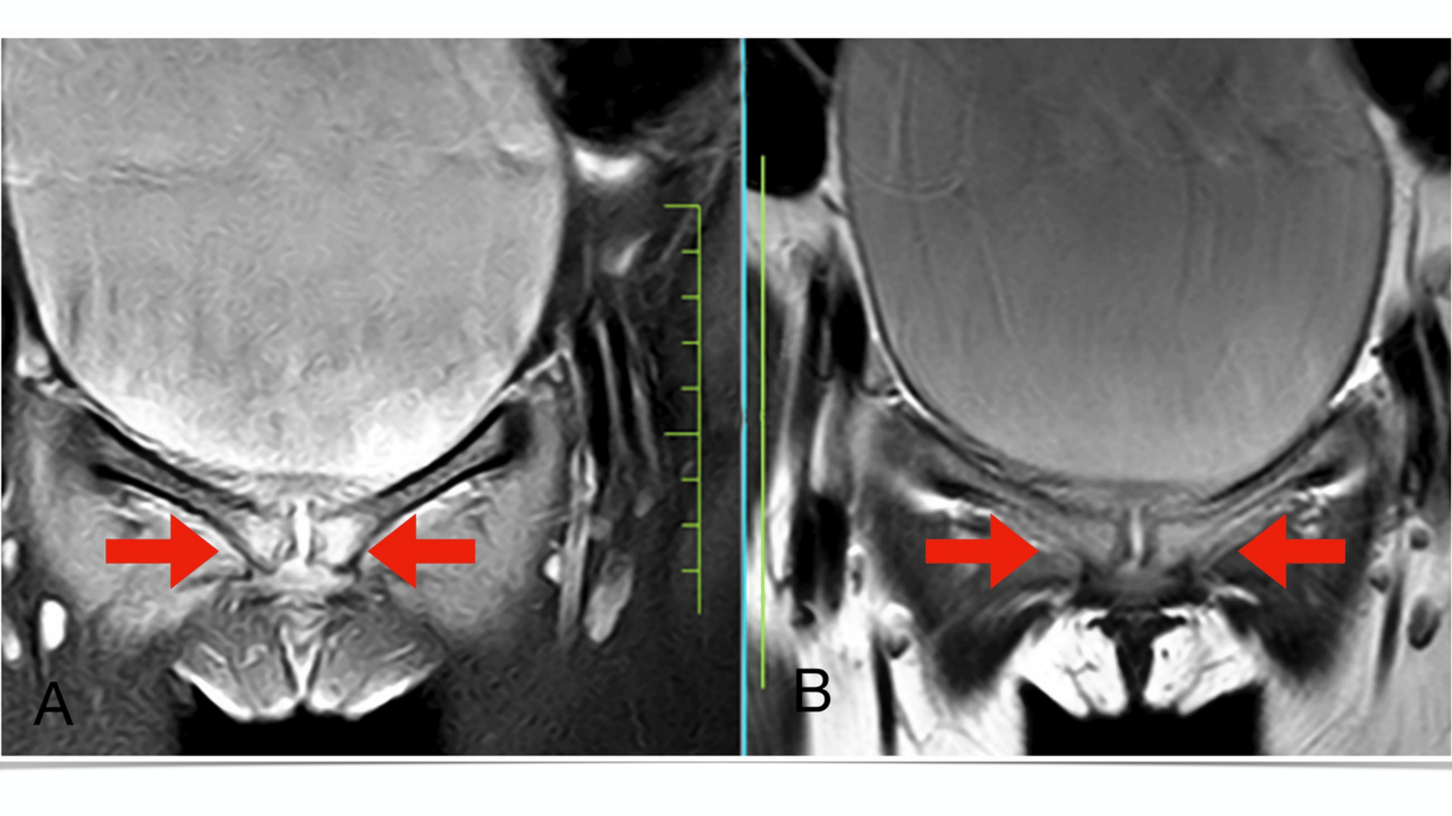
Magnetic resonance imaging coronal section of T2/proton density with fat suppression revealing hyperintensity (A) and coronal section of T1-weighted imaging revealing hypointensity (B) with subchondral sclerosis and bony margin irregularities in the bilateral pubic symphysis, more on the right side.

**Figure 3:**
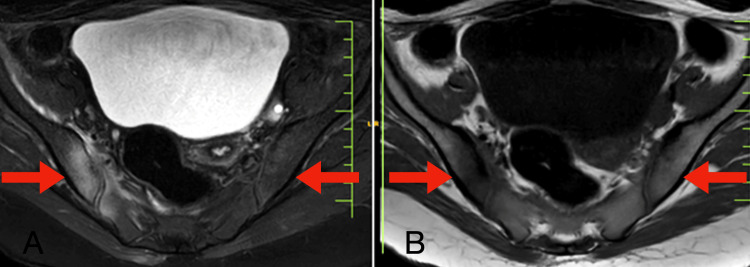
Magnetic resonance imaging axial section of T2/proton density with fat suppression revealing hyperintensity (A) and axial section of T1-weighted imaging revealing hypointensity (B) with subchondral sclerosis and bony margin irregularities in the bilateral sacroiliac joint, more on the right side.

Magnetic resonance imaging investigation revealed T2/proton density with fat suppression hyperintensity with subchondral sclerosis and bony margin irregularities in pubic symphysis (Figure 2) and bilateral sacroiliac joint (Figure 3), more on the right side.

This diagnosis of inflammatory etiology was osteitis pubis with OCI. The patient underwent a six-week physical therapy regimen along with medication. Remarkably, she showed marked improvement within three months with minimal medication usage. Further investigations were conducted, yielding results detailed in (Table 1).

**Table 1:** Laboratory parameters. CBC = complete blood count; Hb = hemoglobin; RBC = red blood cells; PCV = packed corpuscular volume; MCV = mean corpuscular volume; MCH = mean corpuscular hemoglobin; MCHC = mean corpuscular hemoglobin count; RDW-CV = red cell distribution width; TLC = total leukocyte count; CRP = C-reactive protein

Parameter	Result	Reference range
Hb%	10 g/dL	12.5–16
RBC	5.2 mill/mm^3^	4.2–5.4
PCV	31.8%	37–47
MCV	69.0 fL	78–100
MCH	16.6 pg	27–31
MCHC	24.7 g/dL	32–36
RDW-CV	17%	11.6–14
TLC	10,000 cells/mm^3^	4,000–10,500
Neutrophils	55%	44–76
Lymphocytes	43%	20–40
Eosinophils	4%	1–6
Monocytes	5%	2–10
Platelet count	330,000/mm^3^	150,000–450,000
Serum alkaline phosphatase	299 U/L	64-306
RA factor quantitative	5 IU/mL	Up to 20
Serum calcium	9 mg/dL	8.8–10.5
CRP	10 mg/L	Up to 6
Vitamin D3	25.69 ng/mL	Deficiency: <20; insufficiency: 20–<30; sufficiency: 30–100; toxicity: >100

The prognosis of OCI with osteitis pubis is positive, necessitating treatment with calcium, vitamin D, and pain relievers. Surgical intervention is deemed unnecessary due to the self-limiting nature of the condition. Lifestyle modifications were recommended to prevent recurrence, such as avoiding heavy lifting, prolonged bending, and using a lumbar belt for lumbar support. In addition to pharmacological treatment, physiotherapy was advised, and a follow-up visit was scheduled to monitor the patient’s progress.

## Discussion

OCI represents an uncommon cause of persistent lumbar pain with an estimated prevalence of 0.9%-2.5%, predominantly affecting females during the prepartum and postpartum phases [2]. The pathogenesis of OCI remains unclear, although mechanical strain is believed to contribute significantly to its development [5]. While most individuals do not exhibit the human leukocyte antigen B27, there was a previous association between OCI and ankylosing spondylitis. The main characteristic of OCI is the sclerosis observed in the joint portion of the iliac bone. Unlike inflammatory arthritis, OCI typically does not present with heightened inflammatory markers, indicating a lack of association with inflammatory processes [4]. Symptoms of OCI are generally absent; however, in some instances, patients may experience early-onset lower back discomfort resembling axial spondyloarthritis [3]. Pain from OCI often manifests bilaterally and may extend to the posterior thighs and buttocks without specific radiation patterns.

While sacroiliac joint tenderness may or may not be present, the precise pathophysiology of OCI remains inadequately understood. The increased mechanical strain on the ileum is a potential causative factor for OCI in pregnant individuals due to heightened vascularity, leading to bone remodeling and subsequent sclerosis formation. Histopathological examination of sclerosed bone samples typically reveals an excess of lamellar bone in the affected regions [5]. Diagnosis of OCI primarily relies on radiological evidence, necessitating differentiation from spondyloarthropathies, inflammatory arthritis, and malignancies. A distinctive radiological feature distinguishing OCI from other sacroiliac abnormalities is triangular-shaped sclerosis at the iliac border with preserved joint space. Symptoms of osteitis pubis typically manifest one to eight weeks following an inciting incident. These manifestations encompass pelvic discomfort, with potential radiation toward the inner thigh or lower abdominal region, exacerbated by abdominal exertion or movement (notably when assuming an upright position or ascending stairs). Physical examination may reveal tenderness localized over the pubic region, potential indications of joint instability, and, sporadically, the presence of fever [6]. Treatment strategies for OCI typically involve conservative measures such as anti-inflammatory medications and physical therapy, which are commonly effective [7]. The primary objectives of treatment include alleviating pain and stiffness while improving the patient’s quality of life. Educating the patient on the benign nature of the condition and the absence of disease progression based on clinical and radiographic evaluations is the initial step in managing OCI [8]. The primary treatment approach involves rest and pain relief, possibly supplemented with corticosteroids. Surgical intervention, such as curettage, resection, or arthrodesis of the joint, has been employed in rare instances of long-standing pubic osteitis. The prognosis for OCI and osteitis pubis is favorable as the condition does not progress and responds well to conservative interventions. Management often includes rest, physical therapy, and non-steroidal anti-inflammatory drugs. In some cases, corticosteroid/anesthetic injections have been utilized despite the non-inflammatory nature of the disease, with rare instances of surgical core decompression or excision of the affected bone to alleviate symptoms [4].

## Conclusions

Physicians need to have knowledge of OCI and osteitis pubis, a rare condition that typically develops postpartum, with its etiology remaining unidentified. Healthcare providers must distinguish this uncommon, benign, self-limiting ailment from other conditions mimicking sacroiliitis, enabling a prompt and accurate diagnosis to prevent inappropriate treatment and potential adverse effects. The management approach consists of utilizing analgesics and physical therapy, with a focus on lifestyle modifications.
